# Acute atrial wall stretch and the efficacy of flecainide-induced conversion of atrial fibrillation

**DOI:** 10.1007/s12471-015-0661-1

**Published:** 2015-02-20

**Authors:** N.M. van Hemel, J.C. Kelder

**Affiliations:** 1 Department of Cardiology, University Medical Center Utrecht, Heidelberglaan 100, 3584 CX Utrecht, The Netherlands; 2 Department of Cardiology, St Antonius Hospital, Utrecht/Nieuwegein, The Netherlands

Elevated levels of N-terminal pro BNP (NT-proBNP) excreted by stretched cardiomyocytes are attributed to increased cardiac filling pressures arising, for example, in left ventricular relaxation disorders. These conditions elicit higher atrial wall stress which in turn can initiate mechanisms causing atrial fibrillation (AF). However, the presence of AF itself can also produce increased levels of NT-proBNP, as was demonstrated by a drop of the peptide levels when sinus rhythm resumed after converted AF [1]. Not surprisingly, therefore, in the past decade NT-proBNP has received much attention as a potential new marker for AF detection and management [2].

In 2008, Asselbergs and coworkers reported data of the Groningen PREVEND study and showed that newly detected AF could be related to elevated NT-proBNP 4 years earlier at baseline [3]. A new AF-independent predictor was born but its significance was debatable for several reasons [4]. Some studies examined the relation of the duration or time of onset of AF to NT-proBNP levels, whereas other papers demonstrated variable outcomes of the relationship between success rates of AF cardioversion and the peptide levels (see publications of reference [5]). Conflicting results could not promote the role of NT-proBNP as the ideal guide for AF detection or management due to the dubious cause-effect relationship.

The success rate of drug-administered cardioversion of AF depends on many factors such as gender, duration of AF, presence of structural heart disease and so forth. According to our guidelines for drug-administered cardioversion of AF (see www.NVVC.nl), intravenous flecainide, propafenone, ibutilide, or vernakalant are recommended (Class 1, level A) when pharmacological cardioversion is preferred and there is no or minimal structural heart disease. The conversion success rate of flecainide is 67–92 % at 6 hours provided AF is of < 24 hours onset and its safety is very acceptable. On the other hand, spontaneous conversion of recent AF is considerable varying from 66 % in cases of < 24 hours from onset of symptoms of AF in contrast to 17 % of > 48 hours after the onset [6]. Symptomatic < 24 hours AF was shown to be a valid predictor for spontaneous cardioversion, followed by a normal left ventricular function while the size of the left atrium (LA) was of no value [6].

With the purpose to determine the contribution of NT-proBNP to the selection of patients suitable for a successful cardioversion of AF with flecainide, Amin and coworkers carried out a prospective study under very specific clinical circumstances: symptomatic < 24 hours AF, neither structural heart disease, nor left ventricular ejection fraction < 55 %, nor severe comorbidity [3]. Already used antiarrhythmic or other drugs were maintained during the procedure. As expected, a high success rate of conversion (87 %) could be achieved without measurable side effects. In the remaining 13 % of patients without flecainide effect, DC cardioversion was successful: all 112 successively included patients in this study were discharged in sinus rhythm. Of the baseline characteristics, NT-proBNP was the only independent predictor for successful flecainide conversion: at a level < 1550 pg/ml before flecainide treatment a positive predictive value of 94 %, and above this value a negative predictive value of 64 % was computed. The left atrial diameter was not an independent predictor for successful flecainide cardioversion. Surprisingly, the predictive value of NT-proBNP was completely lost at 14-day follow-up (see table 2, [3]). And, also surprising, NT-proBNP levels did not differ in patients with sinus rhythm or AF at 14 days of follow-up (see table 2, [3]).

In our opinion, the message of this study implies no immediate consequences for daily practice for several reasons. The outcome of this study in a select group of patients has to be confirmed by a new study in the same category of patients with comparable methods to comply with the learning-testing method. Secondly, if the validity of the predictor were to be confirmed, it is doubtful whether one would be willing to wait some hours for the laboratory results of NT-proBNP tests before starting flecainide conversion of AF, when faced with a patient with < 24 hours of symptomatic AF. As with ‘time is muscle’ in myocardial ischaemia, duration of AF strongly determines the success of drug-administered cardioversion. Of equal importance: is one sure that the AF profile and cardiac condition of the patient match that presented by this study? And finally, if indeed < 24 hours symptomatic AF is the case, spontaneous cardioversion is a serious option: that worldwide experience can comfort the patient.

However, we have to acknowledge the study by Amin and coworkers because of the observation that different values of NT-proBNP levels of very recent onset AF could be related to the efficacy of flecainide-induced AF cardioversion or not. From a scientific point of view this observation, made in an almost ideal human AF substrate because of absence of confounding factors, counts. Can we explain this finding as an epiphenomenon or does a lower NT-proBNP baseline level truly reflect less acute atrial remodelling of the atria (as well of the ventricles) so that flecainide conversion becomes ‘easier’? Experimental studies can elucidate the causal triangle of effects of acute, longer or maintained elevated atrial and ventricular atrial stress and stretch, the production of NT-proBNP, and the electrophysiological substrate for AF cardioversion respectively. Faced with a patient with < 24 hours symptomatic AF in the emergency room of the hospital, prior to the administration of intravenous flecainide, one can consider reducing the intra-cardiac pressure, as reflected by elevated NT-proBNP, with an intravenous diuretic. This shot can improve the haemodynamic condition for obtaining a successful cardioversion with flecainide. However, this concept also needs to be investigated in prospective randomised studies in AF patients with different baseline characteristics.
